# CypB-CD147 Signaling Is Involved in Crosstalk between Cartilage and FLS in Collagen-Induced Arthritis

**DOI:** 10.1155/2020/6473858

**Published:** 2020-08-29

**Authors:** Qishan Wang, Bingxin Xu, Kaijian Fan, Jing Wu, Tingyu Wang

**Affiliations:** Departments of Pharmacy, Shanghai Ninth People's Hospital, Shanghai Jiao Tong University School of Medicine, Shanghai, China

## Abstract

To investigate the crosstalk between cartilage and fibroblast-like synoviocytes (FLS) in rheumatoid arthritis (RA), we adopted an in vitro coculture system model of collagen-induced arthritis (CIA) cartilage and CIA FLS monolayer. CIA rat samples of the synovium and femur head were collected for isolation of FLS and coculture system. Cartilages were treated with vehicle (Ctrl group), 10 ng/mL interleukin- (IL-) 1*α* (IL-1*α* group), and 10 ng/mL IL-1*α* plus 10 *μ*M dexamethasone (Dex group) for 3 days before coculture with FLS for further 2 days. After the coculture, FLS were collected to determine the influences of articular cartilage on synoviocytes. Whether the CypB-CD147 signaling pathway is involved in the interactions between cartilage and FLS is assayed. Results showed that IL-1*α*-stimulated CIA cartilage promoted the proliferation and reduced the apoptosis of FLS. Increased inflammatory cytokines and decreased p57 expression were found in cocultured FLS stimulated by IL-1*α*-challenged CIA cartilage. Upregulation of NF-*κ*B and I-*κ*B kinase *β* (IKK-*β*) and downregulation of the inhibitor of NF-*κ*B*α* (I-*κ*B*α*) protein were observed in cocultured FLS. After coculture, significant increases in the expression of cyclophilin B (CypB) and CD147 were observed in CIA cartilage and FLS, respectively. Furthermore, results of immunofluorescence staining showed that the anti-CD147 antibody significantly suppressed p65 nuclear translocation in cocultured FLS stimulated by IL-1*α*-challenged CIA cartilage. In conclusion, inflammatory effects in the cartilage-FLS coculture system are associated with the CypB-CD147 mediating NF-*κ*B pathway which may further enhance the inflammation in RA.

## 1. Introduction

Rheumatoid arthritis (RA), a chronic destructive articular synovial inflammatory disease, is featured by articular cartilage degradation and progressive bone erosion caused by synovial compartment inflammation. The proliferating mass of fibroblast-like synoviocytes (FLS) locally invades the articular cartilage and bones and eventually destroys the whole joint. It causes excessive morbidity, mortality, and enormous socioeconomic burdens with an estimated prevalence of 0.5%-1% [1, 2]. Proinflammatory factors secreted by immune cells increase activation, hyperproliferation, and cytokine secretions of RA FLS, and this increase is associated with the severity of articular cartilage degradation [3]. It is well accepted that destruction of the cartilage is mediated by FLS in RA [4, 5]. Synovium-derived inflammatory factors, such as tumor necrosis factor *α* (TNF-*α*), interleukin 1*β* (IL-1*β*), and IL-6, correlate with inflammation-associated cartilage damage by upregulating matrix degradation enzymes [6, 7]. Matrix metalloproteinases (MMPs), induced by IL-1, TNF-*α*, and IL-17, exert an implicit role in the cartilage destruction process [8]. Among synovial products implicated in the process, IL-1 and TNF-*α* work alone or synergistically in stimulation of cartilage to produce MMPs and other degradative mediators, resulting in cartilage loss. Inflamed RA synovium may contribute to cartilage destruction by releasing inflammatory cytokines, leading to dysregulation of chondrocyte function [9]. However, little is known regarding influences of articular cartilage on FLS and whether there is a crosstalk between cartilage and FLS in the pathogenesis of RA. In the present studies, we intend to investigate the influences of articular cartilage on FLS functions and the interplay between them.

CD147, a type I transmembrane protein, is considered a receptor for extracellular cyclophilin A (CypA) and CypB. The Cyp–CD147 interaction modulates inflammatory processes under various disease conditions, including RA [10]. Several reports demonstrated that the CypA/CD147 pathway may be involved in the pathology of RA and treatment with anti-CypA antibody resulted in an intense reduction of MMP-9 production, cartilage erosion, and arthritis in the murine collagen-induced arthritis (CIA) model. Since Cyp/CD147 interaction initiates large quantities of inflammatory signaling cascades, CypA and CypB tend to share some functional similarities in this regard [11]. Although evidence is emerging for CypB in the signaling of CD147, for example, blocking CD147 suppresses CypB-induced cell adhesion, the mechanism of CypB/CD147 interplay in RA remains to be elucidated [12].

Animal models are useful in distinguishing biological changes related to human diseases. CIA animal model shares similar characteristics as that of RA, including synovial hyperplasia, cartilage destruction in inflamed joints and is commonly used for evaluation of physiology and pharmacology in preclinical RA research [13, 14]. As the in vivo gold standard for RA studies, the CIA model is often used in the late, chronic arthritis stage and TNF-*α* and IL-1*β* are important cytokines in rat CIA [15]. Here, to mimic in vivo situation and interactions between the cartilage and FLS in RA, we developed an in vitro CIA cartilage-CIA FLS coculture system. CIA rat samples of the synovium and femur head were collected for isolation of FLS and coculture system to investigate the crosstalk between cartilage and FLS in RA. Our hypothesis is that in CIA rats, inflammatory cytokines (such as IL-1) challenged the cartilage to express MMPs; the elevated MMPs breakdown the matrix of cartilage to release CypB, CypB in turn interacts with CD147 in FLS to stimulate the NF-*κ*B signaling pathway and then activates the expressions of inflammatory cytokines.

## 2. Materials and Methods

### 2.1. Animals

Male Wistar rats, aged 6 weeks, were purchased from Shanghai Slac Animal Center (Shanghai, China) and housed under specific pathogen-free (SPF) facilities in isolated cages. Rats were acclimatized under SPF condition for 2 weeks before induction of CIA model. All animal experiments were approved by Animal Ethics Committee of Shanghai Ninth People's Hospital.

### 2.2. Induction of CIA

Induction of CIA was similar with our previous studies in rats [16]. Bovine collagen II (Chondrex, WA) was emulsified 1 : 1 in incomplete Freund's adjuvant (Chondrex) to form an emulsion. On day 0, rats were injected with the emulsion subcutaneously into the base of rats' tails and a booster was immunized by the same route on day 7. On day 20, hind paw swelling was evaluated to confirm that the CIA models were successfully established.

### 2.3. Isolation and Culture of FLS

The synovial samples of CIA rat models were used for the isolation of FLS as previously described. The synovial tissues were washed three times with phosphate buffer saline (PBS), minced into 1 mm^3^ small pieces, transferred into cell culture flask (Hyclone) and next tiles uniformly. Dulbecco's modified Eagle's medium (DMEM, Hyclone) supplemented with 10% bovine fetal serum (FBS, Hyclone) with 1% bioantibiotics (penicillin and streptomycin, Gibco) was used for liquid-air interface incubation under 37°C and 5% carbon dioxide for 4-6 hours. Furthermore, 10 mL fresh complete culture media were added into flasks and total media was replaced every other day. After 8 days' culture, FLS migrated from the synovial tissues to form a dense FLS monolayer. Then, confluent monolayer FLS were trypsinized, resuspended, and seeded in culture dishes in a density of 5^∗^10^5^/cm^2^. FLS at passages 3-6 were used for experiments in this study.

### 2.4. Coculture of CIA Articular Cartilage and CIA FLS Monolayer

CIA rats were sacrificed 15 days after the second immunization, and articular cartilage explants of bilateral femoral heads were cored using a biopsy punch (10 mm diameter, Fort Lauderdale, FL) and immediately cultured in DMEM supplemented with 10% bovine fetal serum and 1% bioantibiotics until use. Articular cartilage explants were incubated in 10% FBS DMEM for two days to adjust to in vitro setting, and then treated with vehicle (Ctrl group), with 10 ng/mL IL-1*α* (IL-1*α* group, Proteintech), with 10 ng/mL IL-1*α*+10 *μ*M dexamethasone (Dex group) for 3 days before placed into monolayer FLS for another 2 days. Normal and CIA rat cartilages without coculture were pretreated with vehicle, 10 ng/mL IL-1*α*, and 10 ng/mL IL-1*α* plus 10 *μ*M Dex for 3 days before collected for RNA isolation. CIA articular cartilage explants and CIA FLS were cocultured in 48-well plates (Corning, USA) with physical contact. The cocultured FLS and cartilage in the plates were harvested for biological analysis.

### 2.5. Cell Proliferation Analysis

Cell proliferation capacity was assessed by Cell Counting Kit-8 assay (CCK-8, Dojindo, Japan) according to our previous established protocols. After tissue harvest, articular cartilages were pretreated with vehicle, with 10 ng/mL IL-1*α*, and with 10 ng/mL IL-1*α*+10 *μ*M Dex ex vivo for 3 days. Then, monolayer primary FLS were cocultured with pretreated articular cartilage for another 2 days in a 48-well plate. Next, the culture medium was replaced with FLS culture medium containing 10% of the CCK-8 agent. All plates were incubated under 37°C for 3 hours. The absorbance at 450 nm of each well was measured using a microplate reader to assess cell proliferation capacity.

### 2.6. Flow Cytometric Analysis of Cell Apoptosis

For annexin V-APC/propidium iodide (PI) apoptosis analysis, FLS were randomly divided into 3 groups and cocultured with pretreated articular cartilage explant (Ctrl group, 10 ng/mL IL-1*α* group, and 10 ng/mL IL-1*α*+10 *μ*M Dex group) for 48 hours. After rinsing with PBS twice, FLS were digested with 0.25% trypsin, resuspended in binding buffer, and stained with an annexin V-APC/PI (BD, CA, USA) agent according to the manufacturer's protocols.

### 2.7. RNA Isolation and Quantitative Reverse Transcription PCR (qRT-PCR)

After coculture with pretreated articular cartilage, rat cartilages, cocultured FLS and cocultured cartilages were homogenized using TRIzol reagent (Invitrogen) and total RNA was extracted. Then, cDNA was synthesized using cDNA synthesis kit (Takara, Tokyo, Japan) according to the manufacturer's instructions. Real-time PCR was performed with SYBR Green Supermix (Invitrogen, # 111762500) on an ABI 7500 PCR system (Applied Biosystems, CA) following a standard protocol. The primers used were listed in Table 1. The CT value for a specific gene was normalized to that of *β*-actin.

### 2.8. Western Blot Assay

FLS which were cocultured with pretreated articular cartilage were homogenized using sodium dodecyl sulfate (SDS) reagent (Beyotime, Shanghai) supplemented with 1% phosphatase and protease inhibitor cocktail (Thermo Fisher, CN) following the manufacturer's instructions. The protein concentration was assessed with BCA assay kit (Biosharp, China). Next, proteins were separated using electrophoresis, transferred to a polyvinylidene fluoride (PVDF) membrane, blocked with 5% bovine serum albumin (Biosharp, China), and incubated at 4°C with primary antibodies for 16 hours. Then, the PVDF membrane with proteins was incubated with secondary antibodies. Fluorescent signals from protein were detected by Odyssey image system (Lincoln, NE). Primary antibodies used here included NF-*κ*B (p65), inhibitor of NF-*κ*B*α* (I-*κ*B*α*), I-*κ*B kinase *β* (IKK-*β*), CD147, and GAPDH were offered by Cell Signaling Technology (CST, 1 : 1000 dilution). Secondary antibodies used in this study included Anti-mouse IgG (H+L) (DyLight™ 680 Conjugate) and Anti-rabbit IgG (H+L) (DyLight™ 680 Conjugate) were offered by Cell Signaling Technology (CST, 1 : 30000 dilution).

### 2.9. Immunocytochemistry (ICC) Assay

To evaluate the translocation of p65, ICC assay was performed according to the protocol. FLS in the anti-CD147 group were pretreated with 10 *μ*g/mL anti-CD147 monoantibody for 4 hours before coculture with IL-1*α*-induced cartilage and continually given anti-CD147 treatment in two days of coculture setting. Cocultured with pretreated articular cartilage, cocultured FLS were fixed in ice-cold 4% formaldehyde, permeabilized using 0.1% Triton X-100. Next, fixed cells were blocked with 5% BSA and incubated with primary antibody (anti-p65, CST) at 4°C for 16 hours. Furthermore, FLS were incubated with fluorescent secondary antibody (Anti-rabbit IgG (H+L), Alexa Fluor® 594 Conjugate, CST) and counterstained with DAPI. Laser confocal fluorescence microscopy (Leica) was used to detect fluorescent signals from FLS.

### 2.10. Statistical Analysis

Data from all experiments were presented as the means ± SEMs of three independent experiments. One-way or two-way analysis of variance (ANOVA) with Tukey's post hoc test and Student's *t*-test were used for determination of statistical significance. A probability of *P* value less than 0.05 was marked as significant.

## 3. Results

### 3.1. Proproliferation Effect of CIA Cartilage Explants on CIA FLS

**Figure 1:**
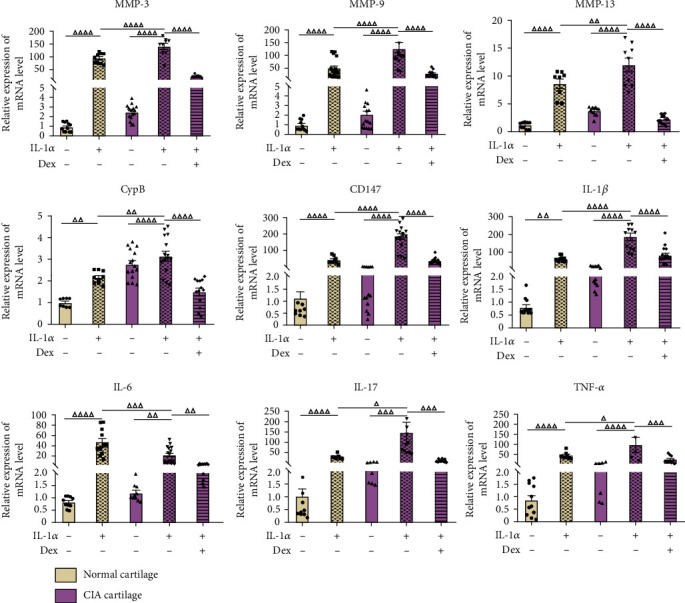
Gene expressions of MMPs, CypB, CD147, and inflammation-related genes in normal and CIA cartilage. Normal cartilage explants were treated with vehicle and 10 ng/mL IL-1*α*, and CIA cartilages were treated with vehicle, 10 ng/mL IL-1*α*, and 10 ng/mL IL-1*α* plus 10 *μ*M dexamethasone, respectively, before collected for total mRNA extraction. All data were presented as a value relative to those in the first group. Values are represented as the mean ± S.E.M. ^△^*P* < 0.05, ^△△^*P* < 0.01, ^△△△^*P* < 0.001, and ^△△△△^*P* < 0.0001 as compared with the indicated group.

As shown in Figure 1, IL-1*α* was able to increase the productions of cytokines (IL-1*β*, IL-6, IL-17, and TNF-*α*), enzymes (MMP-3, MMP-9, and MMP-13), CypB, and CD147 in normal cartilages. In addition, more pronounced effects were observed when cartilage explants from CIA rats were used. Treatment with Dex significantly downregulated the IL-1*α*-induced expression of inflammatory factors, MMPs and CypB, in CIA cartilage. In the upcoming experiments, CIA cartilage will be used in the coculture system with CIA FLS and Dex as the positive control drug to detect the crosstalk between cartilage and FLS.

**Figure 2:**
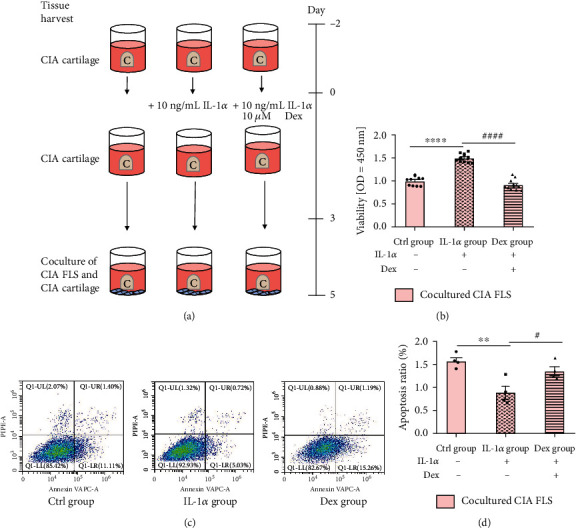
Proproliferation and antiapoptotic effects of CIA cartilage explants on CIA FLS. (a) Schematic of coculture experimental study design. CIA cartilage and CIA FLS were cocultured in 48-well plates with physical contact. Articular cartilage explants were incubated in 10% FBS DMEM for two days to adjust to an in vitro setting and cultured in the presence with vehicle, with 10 ng/mL IL-1*α*, andwith 10 ng/mL IL-1*α*+10 *μ*M Dex for 3 days before being placed into monolayer FLS for another 2 days. (b) Proproliferation effect of CIA cartilage explants on CIA synoviocytes. Cocultured FLS were collected for CCK-8 analysis. (c, d) Antiapoptotic properties of CIA cartilage explants on CIA FLS. Cocultured FLS were harvested for flow cytometric analysis of cell apoptosis. Results are representative images of experiments from at least three independent experiments with similar findings. Values are represented as the mean ± S.E.M. ^∗^*P* < 0.05, ^∗∗^*P* < 0.01, ^∗∗∗^*P* < 0.001, and ^∗∗∗∗^*P* < 0.0001 as compared with the Ctrl group;, ^#^*P* < 0.05, ^##^*P* < 0.01, ^###^*P* < 0.001, and ^####^*P* < 0.0001 as compared with the IL-1*α* group.

The cartilage-FLS coculture system was established as shown in Figure 2(a). After a two-day coculture, the viability assessment was performed on cocultured synovial cells for detection of proliferation. The CCK-8 assay revealed that IL-1*α*-induced CIA cartilage explants prominently promoted CIA FLS viability whereas the addition of Dex substantially restrained it, indicating increased CIA FLS proliferation when FLS were cocultured with CIA cartilage explants treated with IL-1*α* (Figure 1(b), *P* < 0.0001).

### 3.2. Antiapoptotic Properties of CIA Cartilage Explants on CIA FLS

The apoptosis rates of synovial cells in the coculture system were determined by flow cytometry. In contrast with the Ctrl group, the rate of annexin V/PI double-positive cells was reduced in FLS cocultured with cartilage explants treated with IL-1*α* and treatment with Dex elevated cell apoptosis rate (*P* < 0.01; *P* < 0.05). Consistent with our hypothesis, after coculture with IL-1*α*-excited CIA cartilage explants, FLS showed an antiapoptotic effect while Dex could partially reverse it, suggesting an antiapoptotic effect of CIA cartilage explants on CIA FLS (Figure 2(c)).

### 3.3. Effects of CIA Cartilage Explants on Gene Expression Related to Inflammation and Cell Cycle of FLS

**Figure 3:**
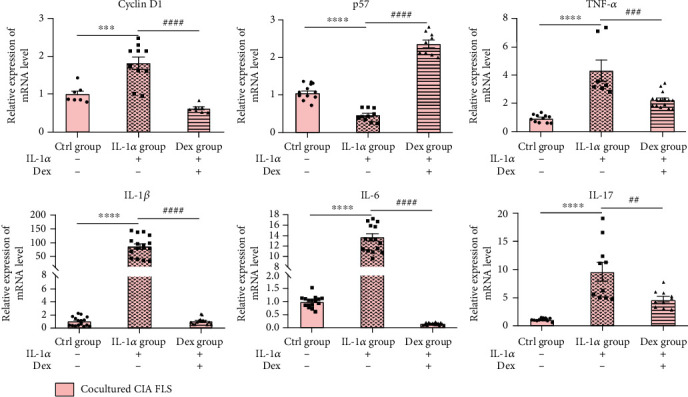
Effects of CIA cartilage explants on gene expressions related to inflammation and cell cycle of FLS. CIA cartilage explants were treated with vehicle (Ctrl group), 10 ng/mL IL-1*α* (IL-1*α* group), and 10 ng/mL IL-1*α* plus 10 *μ*M dexamethasone (Dex group) for 3 days before being placed into monolayer FLS for another 2 days. Cocultured FLS were collected for related gene expressions. All data were presented as a value relative to those in the Ctrl group. Values are represented as the mean ± S.E.M. ^∗^*P* < 0.05, ^∗∗^*P* < 0.01, ^∗∗∗^*P* < 0.001, and ^∗∗∗∗^*P* < 0.0001 as compared with the Ctrl group;, ^#^*P* < 0.05, ^##^*P* < 0.01, ^###^*P* < 0.001, and ^####^*P* < 0.0001 as compared with the IL-1*α* group.

To analyze the effects of CIA cartilage on inflammation and cell cycle of FLS, we used real-time PCR to detect gene expressions of several major inflammatory cytokines and cell cycle-related proteins of cocultured FLS. Gene expressions of IL-1*β*, IL-6, IL-17, TNF-*α*, and Cyclin D1 were increased and mRNA levels of p57 expression was decreased in FLS cocultured with CIA cartilage explants treated with IL-1*α*, while treatment with Dex significantly altered the expression patterns of the above genes (Figure 3). These results showed that CIA cartilage played an indispensable role in gene expressions related to inflammation and cell cycle of CIA FLS.

### 3.4. Effects of CIA FLS on Gene Expressions of MMPs and CypB of CIA Cartilage Explants

**Figure 4:**
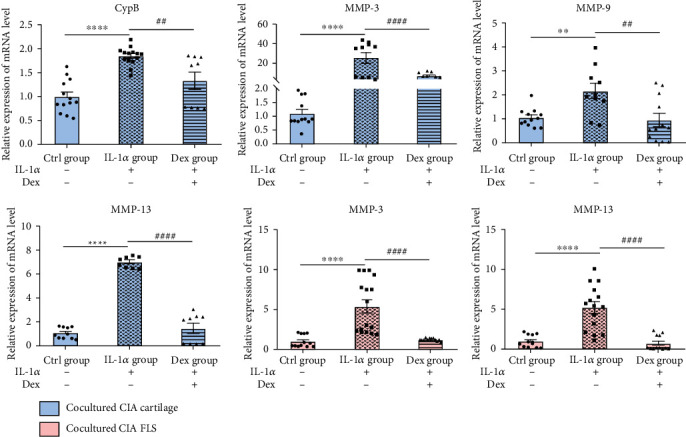
Effects of CIA FLS on gene expressions associated with MMPs and CypB of CIA cartilage explants. All data were presented as a value relative to those in the Ctrl group. Values are represented as the mean ± S.E.M. ^∗^*P* < 0.05, ^∗∗^*P* < 0.01, ^∗∗∗^*P* < 0.001, and ^∗∗∗∗^*P* < 0.0001 as compared with the Ctrl group; ^#^*P* < 0.05, ^##^*P* < 0.01, ^###^*P* < 0.001, and ^####^*P* < 0.0001 as compared with the IL-1*α* group (see Figure 2 for other definitions).

Apart from the effects of CIA cartilage on FLS, we detected the effects of FLS on cartilage. After the coculture with FLS for 2 days, CIA cartilage was collected to analyze the expression of genes related to matrix degradation. Gene expressions of MMP-3, MMP-9, and MMP-13 in cartilage explants were upregulated by coculture and IL-1*α* stimulation. After coculture, gene expressions of MMP-3 and MMP-13 in FLS were also upregulated and pretreatment with Dex on the cartilage could inhibit this upregulation. These results demonstrated that the CIA FLS also exerted a vital effect on CIA cartilage in the coculture system and interplay between cartilage explants and FLS was a key to the inflammatory process (Figure 4).

### 3.5. Crosstalk between CIA Cartilage and CIA FLS Is Associated with CypB/CD147-NF-*κ*B Signaling Pathway

**Figure 5:**
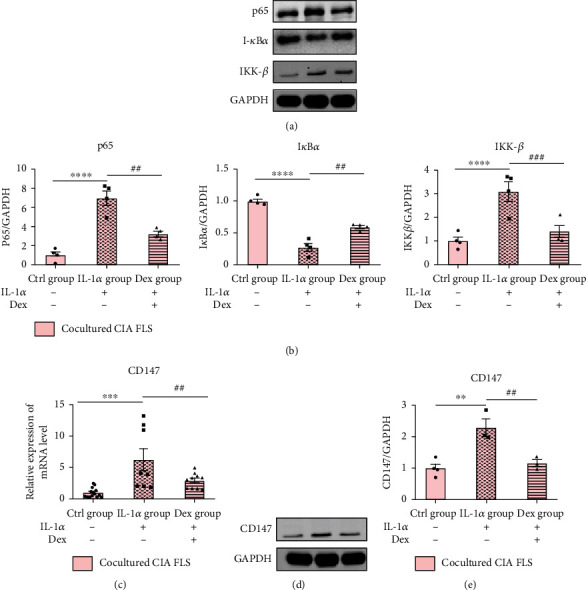
Western blot analysis of the NF-*κ*B pathway and CD147 and gene expression of CD147 from cocultured FLS. (a) Grey value of NF-*κ*B p65, I-*κ*B*α*, and IKK-*β*. (b) A graphic figure of protein expression in the NF-*κ*B pathway. (c) Gene expression of CD147 in three groups of cocultured FLS. (d, e) Western blot analysis of CD147 in all groups of cocultured FLS. Relative expressions of each protein in the Ctrl group were defined as 1. Values are represented as the mean ± S.E.M. ^∗^*P* < 0.05, ^∗∗^*P* < 0.01, ^∗∗∗^*P* < 0.001, and ^∗∗∗∗^*P* < 0.0001 as compared with the Ctrl group; ^#^*P* < 0.05, ^##^*P* < 0.01, ^###^*P* < 0.001, and ^####^*P* < 0.0001 as compared with the IL-1*α* group (see Figure 2 for other definitions).

NF-*κ*B signaling pathway is very important in inflammation; we determined whether NF-*κ*B signaling is activated by CIA cartilage in cocultured FLS. By contrast with the Ctrl group, there were rising NF-*κ*B p65 and IKK-*β* protein levels and declining I-*κ*B*α* protein levels in FLS after coculture with IL-1*α*-challenged CIA cartilage. In contrast, protein levels of NF-*κ*B p65 and IKK-*β* were reduced and I-*κ*B*α* protein levels were upregulated in the Dex group. These findings suggest that inflammatory responses stimulated by CIA cartilage in cocultured FLS may be modulated through the NF-*κ*B pathway (Figures 5(a) and 5(b)).

It was reported that CypB was derived from chondrocytes through induction of MMPs [17]. The interaction between CypB and CD147 could modulate inflammatory processes under various disease conditions [10]. In this study, we explored whether CypB-CD147 is the possible signaling pathways involved in the crosstalk between cartilage and FLS in RA. Results in Figure 4 showed that IL-1*α* could significantly increase the gene expression of MMPs (MMP-3, MMP-9, and MMP-13) and CypB in CIA cartilage explants. Furthermore, results of RT-qPCR and Western blot analysis of cocultured FLS showed that mRNA and protein levels of CD147 were significantly upregulated upon coculture with IL-1-stimulated CIA cartilage (Figures 5(c)–5(e)).

**Figure 6:**
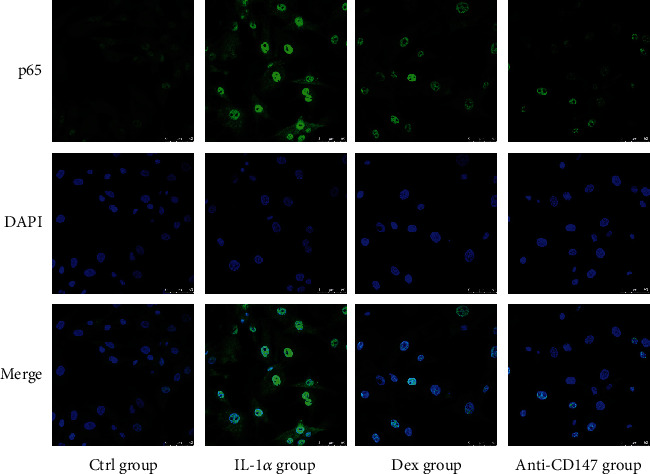
Immunofluorescence staining of p65 nuclear translocation in cocultured FLS from four groups. FLS in the anti-CD147 group were pretreated with 10 *μ*g/mL anti-CD147 monoantibody for 4 hours before coculture with IL-1*α*-induced cartilage, and continually given anti-CD147 treatment in two days of coculture setting. Results are representative images of experiments from at least three independent experiments with similar findings. Values are represented as the mean ± S.E.M; scale bar, 50 *μ*m (see Figure 2 for other definitions).

To investigate whether CD147 regulates NF-*κ*B activity in FLS, anti-CD147 antibody was added in the coculture system and immunofluorescence staining of cocultured FLS was performed. As shown in Figure 6, the nuclear translocation of p65 in cocultured FLS was augmented by CIA cartilage. Moreover, addition of anti-CD147 monoclonal antibody reversed the impact of cartilage and the nuclear translocation of p65 was blocked in the anti-CD147 group. These results elucidated that treatment with anti-CD147 antibody blocks the NF-*κ*B pathway in cocultured FLS, and CD147 displays an indispensable role in the activation of the CD147-mediated NF-*κ*B signaling pathway.

## 4. Discussion

RA is a chronic inflammatory disease that is mainly characterized by symmetrical synovium proliferation and progressive articular cartilage degradation [1]. As a major inflamed site in RA, synovium, mainly composed of FLS, becomes thickened and in the end leads to the formation of invasive pannus. The increased proliferation of activated FLS at the synovium-cartilage interface is a critical event during the formation of an active pannus [6, 18]. Although the major inflammatory target of RA locates in the synovium, articular cartilage destruction is also a leading cause of joint dysfunction [18]. As main effectors of RA, migration and invasion of inflammatory FLS into articular cartilage are essential at the synovium-cartilage interface [6]. FLS also mediate cartilage injury by secreting inflammatory factors, MMPs, and in response, stimulated cartilage explants make various catabolic factors related to the pathogeneses of RA (e.g., TNF-*α*, IL-1*β*, and IL-6). But the effect of cartilage on FLS in RA is barely explored; most of previous studies have been focused on a single-cell or a single-tissue type, such as synoviocytes in RA synovium or synovium culture [16, 19, 20]. However, RA normally exhibits entire joint damage. Therefore, to further explore the interaction of cartilage explants with FLS and the crosstalk between them, we adopted an in vitro coculture system of cartilage explants and FLS monolayer, a model that resembles in vivo RA joints much more closely. This coculture system provides a synovial-cartilage surface that contains ligands into an exchange system, and subsequently cocultured medium that bathes both the cartilage and FLS [21, 22]. Since cartilage cells express IL-1*α* receptor, we chose IL-1*α* as a stimulatory cytokine that maintains the inflammatory state of cartilage. Adopting this coculture setting, we are about to see the influences of IL-1*α*-induced CIA cartilage explants on FLS functions in the coculture system.

Cumulative evidences have confirmed that Dex suppressed inflammation in RA FLS [23]. Dex serves as the first line of medicine in the control of RA for decades with its strong anti-inflammatory and immunosuppressive influences [24]. Dex dampens inflammatory responses in RA by inhibition of inflammatory mediators, like TNF-*α*, IL-1*β*, and IL-6 [13, 23, 25–27]. Zhang et al. noted an in vitro study that activated FLS expressed IL-1*β*, IL-6, and IL-8 and were decreased following exposure to Dex [28]. Dex also has an anticatabolic effect on cartilage in vitro. Low concentrations of Dex were able to protect cartilage explants from TNF-*α* and high dosage suppressed GAG content loss [29]. Black et al. suggested in IL-1*α*-stimulated bovine cartilage explants that Dex treatment largely inhibited transcriptions of IL-6, MMP-3, and MMP-13 [30]. Therefore, in the present study, we chose Dex as our positive medicine to dampen RA in vitro experimental setting. Consistent with these findings, results of CCK-8 assay and flow cytometry suggested that IL-1*α*-activated CIA cartilage promoted FLS proliferation and reduced FLS apoptosis while Dex-treated CIA cartilage restrained FLS proliferation and increased FLS cell apoptosis in the coculture system. In response to CIA cartilage exposure, cocultured CIA FLS possessed proproliferation and antiapoptotic potentials. To delineate the underlying mechanisms, mRNA levels of IL-1*β*, IL-6, IL-17, TNF-*α*, Cyclin D1, and p57 in cocultured FLS were determined. On the one hand, the cell cycle progression is controlled by cyclin, cyclin-dependent kinase (CDK), and CDK inhibitor (CKI). Cyclin D1 and p57 (CKI) both regulate cell cycle at the G1/S checkpoints [31]. An increase in cyclin D1 and a decline of the p57 expression in cocultured FLS stimulated by IL-1*α*-induced CIA cartilage suggested changes in the cell cycle protein expression contributed to the enhanced FLS proliferation [32]. On the other hand, the inflammatory milieu in the articular compartment is mediated by a large complex of cytokine network. Upon being challenged by CIA cartilage, stimulated FLS secreted large amounts of proinflammatory factors, such as IL-1, IL-6, and TNF-*α*, which in turn contributed to the synovial inflammation and cartilage destruction, leading to the progression of RA [33, 34]. IL-17*α* was reported to be one of the upstream inflammatory cytokines in the pathogenesis of RA, which enhanced IL-1 and TNF-*α* production and stimulated synovial cells to become a major source of the production of inflammatory factors [35]. Consistent with our study, stimulated by CIA cartilage, inflammatory gene expressions in the cocultured FLS were all increased in the IL-1*α* group but all mitigated in the Dex group. These observations illustrate that the inflammation process of FLS is affected by CIA cartilage and modulated by a complicated cytokine network.

Also, we noticed remarkable increases in CypB, MMP-3, MMP-9, and MMP-13 in CIA cartilage of the coculture system and the increases in MMP-3 and MMP-13 in cocultured CIA FLS in the IL-1*α*-stimulated group and mitigated changes in the Dex-treated group, indicating effects of CIA synoviocytes on CIA cartilage explants were also unneglectable. CD147-cyclophilin interactions have been demonstrated to play an indispensable role in both in vitro and in vivo studies during RA pathogenesis [36]. Ceuninck et al. have identified the binding sites for CypB on chondrocytes and CpyB was derived from chondrocytes through induction of MMPs [17]. Sharing certain functional similarities with CypA in inflammatory process, CypB might also be responsible for RA cartilage erosion and chondrocyte-derived TNF-*α* is another regulator in mediating aberrant tumor-like behaviors of FLS [6, 11]. In the setting of inflammation, we detected mRNA levels of MMP-3, MMP-9, MMP-13, CypB, CD147, IL-1*β*, IL-6, IL-17, and TNF-*α* in different groups of cartilage explants (normal cartilage with or without IL-1*α* stimulation, CIA cartilage with or without IL-1*α* stimulation, and CIA cartilage treated with IL-1*α* and Dex). As expected, with the induction of IL-1*α*, CIA cartilage showed highest levels among the abovementioned cytokines. From our perspective, IL-1*α*-induced CIA cartilage best mimicked in vivo joint situation of RA patients and CIA cartilage-induced overexpression of CypB might be responsible for synoviocyte inflammation. CpyB was released from chondrocytes by action of MMPs, and elevated MMPs may be one of the mechanisms by which CypB-CD147 interaction contributed to RA progression by aggravating cartilage destruction [17, 36]. Yang et al. reported that NIM811, a cyclophilin inhibitor, reduced MMP-9 secretion in a differentiation process [3]. These results suggest that CIA cartilage may affect inflammation responses of CIA FLS by secretion of CypB, MMPs, and inflammatory factors.

Multiple lines of evidences have indicated that FLS and MMPs contribute to RA perpetuation, and CD147 is regarded as a significant regulator of MMP generation and function [37, 38]. Transmembrane protein CD147, namely, extracellular matrix metalloproteinase inducer (EMMPRIN), has been shown to be expressed predominantly on the FLS membrane in animals with RA [39, 40]. Rich on the surface of synoviocytes, overexpression of CD147 is correlated with the secretion and activation of MMPs, such as MMP-2, MMP-3, MMP-9, and proinflammatory cytokines, such as IL-6 and TNF-*α*, which in turn enhances invasive potential of FLS [7, 36, 39, 41]. Jia et al. conducted an in vivo experiment and reported that CD147/HAb18 mAb attenuated synovial inflammation and cartilage destruction in CIA mice by inhibiting the production of MMPs and proinflammatory mediators (e.g., TNF-*α*, IL-6, and IL-8) and exhibited stronger antierosion effects than the TNF-*α* antibody [40]. Wang et al. confirmed that CD147 was not only an MMP stimulator but also a proangiogenic molecule, and anti-CD147mAb showed stronger antiangiogenic effects than infliximab, thereby suppressing RA development [42]. Studies aimed at CD147 inhibition could be novel and promising strategies for RA treatment. Consistently, we found excessive expression of CD147 mRNA and protein levels in cocultured FLS stimulated by IL-1*α*-induced CIA cartilage whereas decreased the expression in cocultured FLS stimulated by Dex-treated CIA cartilage. It is possible that crosstalk in RA between cartilage and FLS is associated with CypB-CD147 interaction along with stimulation of MMP production. However, the intracellular activation mechanism of upregulated expression of CD147 by CIA cartilage in cocultured FLS during the cartilage-FLS interaction remains unclear.

It is well accepted that NF-*κ*B signaling plays a critical role in various inflammatory diseases. In RA, inflammatory stimulus, like IL-1*β* or TNF-*α*, elicit their effects by inducing NF-*κ*B signaling. During NF-*κ*B signaling activation, the I-*κ*B kinase promotes I-*κ*B*α* phosphorylation and degradation, resulting in the release of NF-*κ*B from the complex and translocation into the nucleus where it turns into a plethora of proinflammatory genes (e.g., IL-6 and TNF-*α*) and upregulates MMPs [43, 44]. This ultimately leads to the progressive destruction of synovium and articular cartilage [45]. To elucidate whether NF-*κ*B signaling pathway was involved in the crosstalk of CIA cartilage and CIA FLS coculture system, we tested protein expressions of p65, I-*κ*B*α*, and IKK-*β* in cocultured FLS. The expression levels of p65 and IKK-*β* had a noticeable upregulation in the IL-1*α* group but were reversed by Dex treatment. Protein content of I-*κ*B*α* was prominently inhibited in the IL-1*α* group but partly restored by the treatment with Dex. These findings suggest that inflammation process stimulated by CIA cartilage in cocultured FLS is at least partially mediated by NF-*κ*B signaling. Moreover, in the present studies, the observation that CD147 activates the NF-*κ*B pathway is correlated with our previous studies [46, 47]. Huang et al. determined that CD147 activated the NF-*κ*B pathway and found a positive feedback loop between CD147 and the NF-*κ*B pathway. Once the NF-*κ*B pathway is activated by CD147, inflammatory cascades activated by NF-*κ*B begins [41, 48]. In our study, induction of IL-1*α*-simulated CIA cartilage in cocultured FLS activated p65 translocation into the nucleus while treatment with anti-CD147 suppressed p65 translocation, illustrating that blockage of CD147 in cocultured FLS inhibited activation of the NF-*κ*B signaling pathway by CD147-mediated p65 translocation.

**Figure 7:**
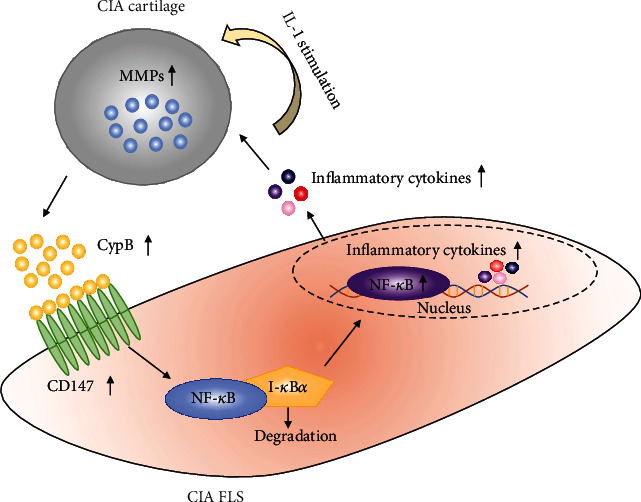
Graphical summary of the crosstalk between cartilage and FLS involving CypB-CD147 signaling in CIA cartilage-synovioctyes coculture system. Upon being challenged by IL-1, CIA cartilage rapidly produces MMPs (e.g., MMP-3, MMP-9, and MMP-13) and releases CypB. As a paracrine proinflammatory cytokine, CypB interacts with CD147 which expresses on the membrane of CIA FLS. Activated by CD147, translocation of NF-*κ*B into the nucleus occurs, followed by a subsequent secretion of inflammatory cytokines, which in turns aggravates the inflammation of cocultured cartilage. Consequently, a vicious circle loop starts.

In conclusion, our findings presented in this manuscript illustrated that inflammatory effects of CIA cartilage on CIA FLS in this coculture system is associated with the CD147-mediated NF-*κ*B pathway and showed a feasible inflammatory pathway between cartilage and FLS : CypB/CD147-NF-*κ*B (Figure 7).

## 5. Conclusions

In this manuscript, we illustrated that inflammatory effects of CIA cartilage on CIA FLS in this coculture system is involved with the CD147-mediated NF-*κ*B pathway and showed a feasible inflammatory pathway between cartilage and FLS : CypB/CD147-NF-*κ*B. This newly designed coculture system might guide the optimized study of cartilage-FLS communication in RA.

## Figures and Tables

**Table 1:** Sequences of specific primers used in RT-qPCR.

Primers	Sequences	
	Forward	Reverse
*β*-Actin	5′-ACGGTCAGGTCATCACTATCG-3′	5′-GGCATAGAGGTCTTTACGGATG-3′
Cyclin D1	5′-TCAAGTGTGACCCGGACTG-3′	5′-CACTACTTGGTGACTCCCGC-3′
p57	5′-GGGCCTCTCATCTCTGACTTC-3′	5′-GTTCTCTCTGGCCGTTAGGC-3′
MMP-3	5′-ATCCCTCTATGGACCTCCCAC-3′	5′-AACAAGACTTCTCCCCGCAG-3′
MMP-9	5′-AGCCGACGTCACTGTAACTG-3′	5′-AACAGGCTGTACCCTTGGTC-3′
MMP-13	5′-ACCCAGCCCTATCCCTTGAT-3′	5′-TCTCGGGATGGATGCTCGTA-3′
CypB	5′-CTCCGTGGCCAACGATAAGA-3′	5′-AGCCAAATCCTTTCTCTCCTGT-3′
CD147	5′-AAACGACAGCTGCTCCCAG-3′	5′-TTACGATGGTGCCCGGTTC-3′
IL-1*β*	5′-AGCAGCTTTCGACAGTGAGG-3′	5′-CTCCACGGGCAAGACATAGG-3′
IL-6	5′-CTCTCCGCAAGAGACTTCCAG-3′	5′-TTCTGACAGTGCATCATCGCT-3′
IL-17	5′-CCATCCATGTGCCTGATGCT-3′	5′-GTTATTGGCCTCGGCGTTTG-3′
TNF-*α*	5′-ATGGGCTCCCTCTCATCAGT-3′	5′-GCTTGGTGGTTTGCTACGAC-3′

Note. MMP-3: matrix metalloproteinases-3; IL-1*β*: interleukin-1*β*; TNF-*α*: tumor necrosis factor-*α*.

## Data Availability

All the data supporting the findings were shown within the paper and can be requested from the corresponding author.
